# Effects of Aging and Adult-Onset Hearing Loss on Cortical Auditory Regions

**DOI:** 10.3389/fnins.2016.00199

**Published:** 2016-05-11

**Authors:** Velia Cardin

**Affiliations:** ^1^Department of Experimental Psychology, Deafness, Cognition and Language Research Centre, University College LondonLondon, UK; ^2^Department of Behavioural Sciences and Learning, Linnaeus Centre HEAD, Swedish Institute for Disability Research, Linköping UniversityLinköping, Sweden

**Keywords:** hearing loss, aging (aging), aging and cognitive function, cognitive decline, Auditory cortex, humans

## Abstract

Hearing loss is a common feature in human aging. It has been argued that dysfunctions in central processing are important contributing factors to hearing loss during older age. Aging also has well documented consequences for neural structure and function, but it is not clear how these effects interact with those that arise as a consequence of hearing loss. This paper reviews the effects of aging and adult-onset hearing loss in the structure and function of cortical auditory regions. The evidence reviewed suggests that aging and hearing loss result in atrophy of cortical auditory regions and stronger engagement of networks involved in the detection of salient events, adaptive control and re-allocation of attention. These cortical mechanisms are engaged during listening in effortful conditions in normal hearing individuals. Therefore, as a consequence of aging and hearing loss, all listening becomes effortful and cognitive load is constantly high, reducing the amount of available cognitive resources. This constant effortful listening and reduced cognitive spare capacity could be what accelerates cognitive decline in older adults with hearing loss.

## Introduction

Normal aging in humans is often accompanied by hearing loss (Lin et al., 2011b; Humes et al., 2012). This age-related hearing loss in known as presbycusis. In the UK alone, 6.4 million (60%) of those over 65 years of age have some hearing loss (Action-on-Hearing-Loss, 2011)—when compared to 25 year-olds, 70 year-olds have average hearing thresholds that are raised 10 dB at lower frequencies (250–1000 Hz), and 20–60 dB at higher frequencies (2–8 kHz). Even though pure-tone detection is strongly associated with auditory processing deficits (Humes et al., 1994; Humes, 1996), the relationship between real-life auditory processing and pure-tone sensitivity is weak, and elderly adults with similar thresholds vary in their ability to understand speech in noise (Schneider et al., 2002; Humes, 2007; Wilson and McArdle, 2008). Other perceptual variables, such as temporal and intensity discrimination, frequency resolution, audibility and binaural processing, account for some of the differences, but they cannot explain all the observed variance (Glasberg et al., 1984; Moore and Peters, 1992; Moore et al., 1992; Sommers and Humes, 1993; Pichora-Fuller and Schneider, 1998; Schneider and Pichora-Fuller, 2000; Gordon-Salant, 2005; Grose et al., 2006; Souza and Boike, 2006; Humes et al., 2010; Gordon-Salant et al., 2011; Grose and Mamo, 2012; Tun et al., 2012). Evidence also suggests that older adults with hearing loss have poorer speech comprehension than older adults with better hearing (Stewart and Wingfield, 2009; Adank and Janse, 2010; Tun et al., 2010), and than young adults with equivalently poor hearing (Dubno et al., 1984; Fitzgibbons and Gordon-Salant, 1995; Wingfield et al., 2006b). Older adults are also more likely to have “hidden hearing loss,” where there is damage in high-threshold auditory nerve fibers (usually as a consequence of noise exposure, Kujawa and Liberman, 2009; Schaette and McAlpine, 2011; Plack et al., 2014; Viana et al., 2015). This cochlear neuropathy is not reflected in conventional audiograms, but affects auditory processing at all subsequent levels, including cortical responses (Bharadwaj et al., 2015). Together, these pieces of evidence suggest a functional interaction between the effects of hearing loss and aging, exacerbating the effects that each has in isolation.

It is also known that as a consequence of aging there are changes in brain structure, including reductions in white matter integrity, gray matter volume and thinning of the cortex (Sullivan and Pfefferbaum, 2006; Grady, 2012; Onoda et al., 2012; Betzel et al., 2014). These are accompanied by changes in connectivity between functional networks, and recruitment of additional brain regions for performance of several tasks (see for a review Grady, 2012; Park and McDonough, 2013; Bennett and Madden, 2014; Lockhart and DeCarli, 2014). It is still debatable whether this reflects compensation, dedifferentiation (i.e., loss of functional specialization), or less efficient use of neural resources (see Grady, 2012, for a review). These neural changes have an impact on behavior, with decline in several cognitive domains including attention, working memory and processing speed (Deary et al., 2009; Tun et al., 2012). Cognitive abilities, in particular working memory, are strong predictors of successful understanding of speech in noise, and a decline in these is likely to impact negatively on auditory and speech processing (Pichora-Fuller, 2003; Akeroyd, 2008; Arlinger et al., 2009; Anderson et al., 2013; Arehart et al., 2013; Zekveld et al., 2013). Importantly, not all brain regions are equally affected by older age, and some functions are preserved through the lifespan. For example, structural changes are more pronounced in the prefrontal cortex (Raz et al., 1991), connectivity in the default-mode network seems to be particularly affected in older age (Tomasi and Volkow, 2012), and even though many cognitive functions decline, language comprehension skills are preserved in older adults (Shafto and Tyler, 2014).

Evidence suggests that hearing loss in older adults also contributes independently to cognitive decline, exacerbating the effects of physiological aging (Lin et al., 2011a, 2014; Pichora-Fuller and Levitt, 2012; Wayne and Johnsrude, 2015). Several theories have been put forward to explain the relationship between hearing loss and cognitive decline in the elderly (Baltes and Lindenberger, 1997; Pichora-Fuller, 2003; Lindenberger and Ghisletta, 2009; Sarampalis et al., 2009; Tun et al., 2009; Heinrich and Schneider, 2010; Ronnberg et al., 2011). However, it is not yet clear what the relationship between them is, and which neural mechanisms are affected.

This review provides a summary of the effects of adult-onset hearing loss and aging on the function and structure of the central auditory system in humans. The exclusion of literature investigating early onset hearing loss is because the effects of hearing loss on brain structure and function will vary with the developmental stage and biological age at which the sensory deprivation occurs. This is mainly due to the following reasons:

1. Hearing loss causes deficits in speech perception and human communication (see for examples Dubno et al., 1984; Waldstein, 1990; Briscoe et al., 2001). The impact of hearing loss in communication is going to be sizeable at any point in life, but it will also hinder spoken language acquisition if it occurs during infancy (Newport, 1990; Blamey et al., 2001; Nicholas and Geers, 2006; Lyness et al., 2013). Therefore, prelingual and postlingual hearing loss cannot be equated.
2. Early in life there are “sensitive periods” of enhanced neural plasticity, in which the development of neural systems is highly modulated by environmental experience (Hensch, 2004). As a consequence, auditory deprivation that coincides with developmental sensitive periods could result in different patterns of neural reorganization than those that occur later in life. This is not only the case during infancy, but also during adolescence, through which the brain continues developing (Fuhrmann et al., 2015).

In short, due to the interplay that hearing loss will have with language acquisition and with sensitive periods of neural plasticity in early life, the effects of adult-onset hearing loss cannot be equated to those of onset during adolescence or infancy (Lyness et al., 2013). Therefore, the studies reviewed here will look exclusively at adult-onset hearing loss, with the aim of understanding what the effect of hearing loss are in a brain that has established sensory and cognitive systems.

The review is divided into two main sections:

1. Brain structural changes as a consequence of hearing loss and aging, where a short description of the structure of the human auditory cortex is presented, followed by a review of morphometry and diffusion MRI studies.
2. Effects of aging and hearing loss on brain function, where evidence from fMRI and EEG studies is reviewed, revealing the interaction between aging and hearing loss in cortical function.

This paper concentrates on evidence obtained from the study of humans, given the relevance that language function has for human communication, but also for auditory processing. However, animal studies have been extremely informative for our understanding of hearing loss and aging, and several excellent reviews discuss these topics in detail (Frisina, 2009; Fetoni et al., 2015; Ouda et al., 2015).

## Brain structural changes as a consequence of hearing loss and aging

### Auditory cortex in humans

In non-human primates, primary auditory areas are grouped in a “core” region, and secondary areas are grouped in “belt” and “parabelt” regions, located concentrically around the core (see Hackett, 2011, for a review). The core regions represent the first level of cortical auditory processing, and the surrounding belt and parabelt regions support higher levels of processing. In humans, the auditory cortex is located in the superior temporal gyrus (STG), but its precise extent and borders are not clear (Hackett, 2015). The auditory core is located in Heschl's gyrus (HG), but its functional subdivision is still a matter of debate (e.g., Formisano et al., 2003; Da Costa et al., 2011; Dick et al., 2012). An alternative anatomical approach is to characterize primary auditory areas based on their microstructural properties. Post-mortem cytoarchitectonic analysis has revealed three distinct areas in Heschl's gyrus (from postero-medial to antero-lateral): Te1.1, Te1.0, and Te1.2. Based on its granularity, Te1.0 is the most likely human homolog of the primary auditory cortex (Morosan et al., 2001; Tahmasebi et al., 2009; Hackett, 2011). The fact that these cytoarchitectonic definitions bypass auditory stimulation for the definition of functional areas, which can be complicated or not possible in participants with auditory deficits, have made these cytoarchitectonic maps popular in the study of hearing loss in humans.

### Morphometry

Techniques measuring gray matter volume, cortical thickness and surface area have been the most commonly used to assess structural brain changes as a consequence of hearing loss (Table 1). However, outcomes from these techniques have been mixed. Of those studies measuring morphometric changes in auditory cortices, two have showed a positive correlation between hearing loss and reductions in gray matter volume (Peelle et al., 2011; Eckert et al., 2012), whereas other two did not find an effect (Boyen et al., 2013; Profant et al., 2014). These conflicting findings could be explained by the specificity in the definition of the regions of interest. Eckert et al. (2012) and Peelle et al. (2011), who found that hearing loss is associated with gray matter loss, used probabilistic cytoarchitectonic maps which are more likely to contain exclusively primary auditory regions. Instead, Profant et al. (2014) and Boyen et al. (2013) measured structural changes in the whole of Heschl's gyrus, which contains not only primary auditory regions, but also other functionally and anatomically distinct areas, and for this reason results are deemed to be more heterogeneous. These differences suggest an association between hearing loss and gray matter that may be constrained to primary auditory cortices. They also call for specificity when defining auditory regions of interest in future studies, as averaging neuroimaging measurements across a whole gyrus may hinder true effects in discrete regions.

**Table 1:** **Studies evaluating the effect of hearing loss on the structure of the human brain**.

**Study**	**Technique**	**Hearing loss (db HL)**	**Mean age in years (range)**	**Results**
Boyen et al., 2013	Morphometry	HI + T (41) HI (45) NH (–)	HI + T: 56 (31–75) HI: 63 (44–84) NH: 58 (50–69)	HI: ↑ GM in STG, MTG; ↓in SFG, occipital lobe and hypothalamus
Eckert et al., 2012	Morphometry	Adults variable hearing loss (normal hearing to profound loss)	69.6 (54–88)	↓ GM volume associated with high-frequency hearing loss in auditory regions, particularly in left te1.0
Husain et al., 2011	dMRI	HI (25) HI + T (28) NH (12)	HI: 51 (31–64); HI + T: 56 (42–64); NH: 48 (32–63)	HI: ↓ FA in right superior and inferior longitudinal fasciculi, corticospinal tract, inferior fronto-occipital tract, superior occipital fasciculus, and anterior thalamic radiation.
	
	Morphometry			HI: ↓ GM STG, ACC and superior and medial frontal gyrus
Lin et al., 2008	dMRI	HI (> 90) NH (< 25)	HI: 32 (–)^*^ NH: 31 (–)	HI: ↓ FA and ↑ radial diffusivity in LL and IC
Lin et al., 2014	Morphometry	NH (< 25) HI (> 25)	NH: 67 (56–86) HI: 74 (56–86)	Hearing impairment accelerated volume loss in whole brain and regionally in right temporal lobe
Peelle et al., 2011	Morphometry	Adults variable hearing (21)	66 (60–77)	Correlation between hearing and GM volume in primary auditory cortex (Te1.0 + Te11).
Profant et al., 2014	dMRI	NH (< 20) Mild presbycusis (< 20 at 2 kHz; 75 at 12.5 kHz) Expressed presbycusis (35 at 2 kHz; 80 at 12.5 kHz)	NH: 25 (–) Mild presbycusis: 68 (–) Expressed presbycusis: 70 (–)	Tendency hearing loss effect in MD in white matter underneath HG
	Morphometry			No effect of hearing loss
Yang et al., 2014	Morphometry	NH (< 25) Unilateral hearing loss (> 40)	NH: 54 (41–60) Unilateral hearing loss: 54 (41–60)	HI: ↓ GM in bilateral PCG and precuneus; left STG, MTG, ITG; right parahippocampal gyrus and lingual gyrus

*dMRI, diffusion magnetic resonance imaging; HI, hearing impaired; HI + T, hearing impaired with tinnitus; NH, normal hearing; GM, gray matter; STG, superior temporal gyrus; MTG, middle temporal gyrus; SFG, superior frontal gyrus; FA, fractional anisotropy; ACC, Anterior cingulate cortex; LL, lateral lemniscus; IC, inferior colliculus; HG, Heschl's gyrus; PCG, posterior cingulate gyrus; ITG, inferior temporal gyrus; (–), Information not provided*.

* *Age range is not provided in this study. It is possible that adolescents have been included in the sample, as authors only specify recruiting participants older than 8 years of age*.

The effects of hearing loss on the morphometry of other structures of the temporal lobe and the rest of the brain are also mixed (see Table 1). Boyen et al. (2013) found an increase in gray matter volume in STG and middle temporal gyrus (MTG) in hearing impaired individuals. Instead, Husain et al. (2011) found reductions in gray matter in STG in those with hearing loss, but not in those with hearing loss and tinnitus, and Yang et al. (2014) reported reduced gray matter in STG, MTG and inferior temporal gyrus in patients with unilateral hearing loss. Similar discrepancies are found when looking at results from the whole brain (Table 1).

Regarding the effect of aging on the morphometry of auditory regions, whole brain analyses not always show differences between young and older adults in temporal lobes, but those that define discrete regions of interest do. Reductions in gray matter volume and cortical thickness as a consequence of aging have been found in Heschl's Gyrus, planum temporale, and STG (Harris et al., 2009; Tremblay et al., 2013; Meunier et al., 2014; Profant et al., 2014). These effects are not uniformly present across the brain (Profant et al., 2014), suggesting that they are indeed specific to auditory areas, and not general senescent effects.

In trying to understand discrepancies in the observed effects of hearing loss and aging on brain morphometry, there are three salient factors: (1) Lack of specificity when defining regions of interest, as explained above (e.g., the occipital lobe has many functionally specialized areas); (2) Measuring the effects of high frequency hearing thresholds vs. average pure tone thresholds. Hearing loss tends to be greater for higher-frequency sounds, and as discussed by Eckert et al. (2012), using the high-frequency component of hearing thresholds may provide more accurate estimations of the effects of hearing loss on neural structure; and (3) All the studies mentioned above are cross-sectional (typically N ≈ 20). Cross-sectional and longitudinal studies do not always provide concordant evidence. For example, in the neurobiology of human aging, longitudinal changes are not always reflected in cross-sectional analyses, and estimated rates do not match longitudinal measurements (Raz et al., 2005; Raz and Lindenberger, 2011). In the field of hearing loss, this discrepancy between cross-sectional and longitudinal measurements becomes apparent in the study by Lin et al. (2014), in which the authors compared brain volume in hearing impaired (*N* = 51) and normal hearing (*N* = 75) older adults in a baseline scan and a follow-up scan (on average) 6.4 years later. They found no significant differences between the groups at the baseline measure, but after the follow-up scan those with hearing impairment had an accelerated volume decline in the whole brain, and particularly in the right temporal lobe. In short, the use of more specific, hypothesis-driven definitions of regions of interest, in combination with longitudinal approaches, could aid some light on the mixed effects found when measuring gray matter changes as a consequence of hearing loss.

### Diffusion MRI studies

Another non-invasive brain imaging technique to study brain structure is diffusion MRI (dMRI). This technique measures microstructural parameters, including fractional anisotropy (FA) and mean diffusivity (MD), which reflect properties such as the degree of density and orientation dispersion of neuronal fiber bundles (Jones, 2008; Johansen-Berg and Rushworth, 2009). It also allows tracing of anatomical connections in the living brain.

Microstructural changes associated with hearing loss have been found in subcortical components of the auditory pathway [reduction in fractional anisotropy (FA) and increase in radial diffusivity], such as the lateral lemniscus and the inferior colliculus (Lin et al., 2008). White matter tracts underneath Heschl's gyrus also show a tendency for an effect in microstructure (increase in AvgL2L3, which the authors tentatively suggest could reflect demyelination), but differences do not achieve statistical significance (*N* = 12–15 per experimental group; Profant et al., 2014). In a whole-brain analysis, a reduction in FA was also found in a large cluster of the right hemisphere which comprised the corticospinal tract, inferior and superior longitudinal fasciculi, inferior fronto-occipital fasciculus, superior occipital fasciculus and anterior thalamic radiations (Husain et al., 2011).

Compared to younger adults, older adults show reductions in FA in the acoustic radiation, Heschl's gyrus and STG (Lutz et al., 2007). (A non-significant trend in this direction is also observed in the white matter under Heschl's gyrus in the study of Profant et al., 2014).

The evidence so far is scarce, but it suggests that hearing loss and aging result in microstructural changes in white matter tracts of the auditory pathway, potentially compromising cortical auditory function.

## Effects of aging and hearing loss on brain function

### fMRI studies

One of the most common tools for the study the human brain is functional Magnetic Resonance Imaging (fMRI). By detecting changes in blood flow that occur as a consequence of neural activity, this technique allows indirect measurement of brain function in a non-invasive manner and with a spatial resolution of millimeters (Ogawa et al., 1990). fMRI has been widely used in the study of aging and hearing loss, but not without challenges. Specifically, there are high levels of acoustic noise during MRI scanning, and special efforts have to be put into selecting acquisition sequences and their interaction with auditory stimuli (Peelle, 2014). Age also produces changes in the vasculature, affecting the blood-oxygenation level-dependent (BOLD) signal, and it is important to separate these vascular effects from those that arise as a consequence of differences in neural function between young and older adults (Tsvetanov et al., 2015). However, some of the most relevant issues affect all studies of auditory processing in aging and hearing loss. Stimuli selection is one of these—the use of simple acoustic stimulation (e.g., detection of tones) may hinder effects that are only evident when listening in challenging conditions, whereas complex tasks may reflect problems in cognition, and not auditory processing *per se*. Furthermore, as explained in more detail in the following section, differences in cortical effects between groups and conditions may be due to compromised processing in peripheral and subcortical regions, which will affect the quality of the signal that arrives to the cortex. In addition, hearing loss is common in older adults, and auditory thresholds are not always measured in studies of aging. Consequently, effects that are assigned to aging may be due to concomitant hearing loss. This confound is sometimes avoided by using stimuli with the same audibility for all participants, but this can compromise frequency encoding. When reviewing the evidence below, these issues will be highlighted when it is likely that they can affect the interpretation of results.

fMRI studies of auditory processing show less activity in cortical auditory regions in older than younger adults. This has been demonstrated using a variety of paradigms, from those in which participants passively listened to words (Cliff et al., 2013), to speech in noise tasks (Hwang et al., 2007; Wong et al., 2009; Bilodeau-Mercure et al., 2015; Manan et al., 2015). This is contrary to findings using simple acoustic stimuli, where there is an increase in the level of activity observed in auditory cortex as a function of age (Profant et al., 2015).

The effects of hearing loss on the function of cortical and subcortical areas also vary with the complexity of the stimuli. Boyen et al. (2014) showed a negative correlation between hearing thresholds (mean PTA = 43 dB HL) and activation elicited by acoustic stimulation in subcortical structures of the auditory pathway (medial geniculate body, inferior colliculus and cochlear nucleus). Such a relationship was not found in the STG. Differences in cortical activation between older adults (mean 69 years) with mild (8 kHz PTA ~30 dB) and expressed (8 kHz PTA ~70 dB) presbycusis were also absent in the study of Profant et al. (2015). However, both these studies used basic auditory stimulation. In a study of sentence comprehension in older adults (mean = 64.9 years), Peelle et al. (2011) showed that hearing ability correlated with activity not only in subcortical regions, but also in the auditory cortex (STG encompassing also primary auditory regions, but without defining them specifically). As mentioned above, they also showed that hearing thresholds were positively correlated with gray matter loss in cytoarchitectonic regions Te1.0 and Te1.1, providing a structural and functional link, and suggesting that cortical differences may only be apparent when using fMRI with complex auditory stimulation and challenging tasks. This link between structure and function is important, because cortical functional effects can always reflect processing deficiencies or compensatory mechanisms from subcortical or peripheral stages. However, when functional effects are linked to structural damage or atrophy, it suggests cortical mechanisms are indeed compromised.

Reductions in evoked fMRI activity and in gray matter volume in auditory areas, in particular the STG, are often accompanied by differential recruitment of other cortical regions. For example, in a study of speech in noise, Wong et al. (2009) found that the reduction in activity in auditory areas of older individuals (mean age = 68 years; range = 63–75) was accompanied by stronger recruitment of parietofrontal regions, and that this additional recruitment correlated with performance. In a further study (Wong et al., 2010), they showed that the volume of the left pars triangularis and the cortical thickness of the left superior frontal gyrus were positively correlated with performance in a speech-in-noise test (mean age = 67 years; range = 62–75 years). Gray matter volume in left auditory cortices has also been found to be positively associated with word recognition skills, and negatively associated with activation in anterior cingulate cortex (ACC; age range = 19–39 and 61–79 years; Harris et al., 2009) and middle frontal gyrus (mean age = 42.1 years; range = 21–79 years; Eckert et al., 2008). Furthermore, Tyler et al. (2010) demonstrated that, during a word monitoring task, older adults (mean age = 67.4 years; range = 49–86 years) show additional recruitment of frontal right hemisphere regions. This additional recruitment was positively associated with the level of gray matter atrophy in left frontotemporal regions, including STG, and aided older adults in performing at the same level than the younger group. Summarizing, as a consequence of aging and hearing loss, there are morphological changes in auditory areas, which are consistent with structural damage. It is not known whether this damage is the cause of compromised auditory processing, and the reliance on more cognitive resources to aid perception. What it is clear from the evidence reviewed above, is that additional recruitment of frontal regions is observed when there is damage in auditory areas, and that the amount of damage in temporal cortices and the recruitment of frontal regions predict behavioral performance.

The additional activation and recruitment of frontal regions during auditory tasks, observed both as a consequence of aging and hearing loss, is likely to reflect more widespread changes in network dynamics. The connectivity of cortical functional networks changes in older age. In younger adults, functional activity in the left STG is positively correlated with activity in the right STG; in older adults there is no significant correlation between activity in the left STG and the right STG, but activity in the left STG is significantly correlated with activity in a more spread set of areas, including frontal regions (mean age = 67 years; range = 63–75) (Wong et al., 2009). Studies of aging have also shown reduced connectivities within a sentence-comprehension network (Peelle et al., 2011; see Table 1), and between the salience network and the auditory network (Onoda et al., 2012). This latter effect comes from a resting state study in which connectivity was correlated with age (*n* = 73; mean age = 60; range = 36–86), and it is not clear whether it is mediated by age itself or by age-related hearing loss (Onoda et al., 2012), as hearing thresholds were not measured. In a study evaluating changes in functional connectivity as a consequence of hearing loss (mean 36 dB HL at 4 kHz), Husain et al. (2014) did not find evidence of hearing loss affecting the pattern of functional connectivity between auditory regions and other cortical areas. However, hearing loss affects the pattern of connectivity in the attention and default mode networks (Husain et al., 2014), suggesting that the effects of aging could be at least partly due to concomitant hearing loss. Importantly, the level of network reorganization observed in older adults is associated with the level of gray matter loss in temporal regions rather than age itself (Meunier et al., 2014). This provides more indirect evidence to support the idea that the effects of aging on the reorganization of cortical functional networks are at least partially due to age-related hearing loss, given that hearing loss is associated with gray matter reductions in temporal areas (see above).

Dynamics of the salience network seem to be particularly influenced by aging and hearing loss. This network includes the ACC, the pre-supplementary motor area and the insula, and it is generally thought to be involved in the detection of salient events, and in deploying the appropriate behavioral responses to these events (Menon and Uddin, 2010). Functional recruitment of components of the salience network during auditory tasks changes in older age. During speech perception tasks, younger adults show stronger activations of ACC in incorrect trials compared to correct ones (Sharp et al., 2005; Harris et al., 2009), and while listening to degraded speech more than when listening to clear speech (Erb et al., 2013). Instead, in older adults (mean age = 71; range = 61–79 years), there is higher overall activity in ACC during speech perception (Harris et al., 2009), and similar levels of activations with degraded and clear speech (Erb and Obleser, 2013). Furthermore, ACC recruitment is negatively associated with word recognition and speech comprehension in older adults (Sharp et al., 2005; Harris et al., 2009; Erb and Obleser, 2013). Erb and Obleser (2013) argue that it is the degree to which the ACC is engaged and disengaged in degraded and clear speech, respectively, that is associated with better speech comprehension, and that this dynamic range of ACC activity decreases with age, with detrimental consequences for comprehension. Importantly, the level of recruitment of ACC is correlated with gray matter volume loss in HG/STG (Eckert et al., 2008; Harris et al., 2009). Thus, additional cognitive resources are used to achieve successful auditory perception in challenging conditions. In turn, aging and hearing loss affect the successful deployment of such strategies. Future studies should aim to disentangle if this is a direct effect on the mechanisms of cognitive control, or if it is mediated by gray matter volume loss in auditory cortices and compromised auditory processing, as discussed above.

Additional recruitment of frontal regions, in particular in the cingulo-opercular cortex (medial frontal cortex, anterior insula and frontal operculum), aids in adaptive control during word recognition (Vaden et al., 2013, 2015), in which by learning from difficult or error trials, performance improves in following trials. Vaden et al. (2013) have shown that cingulo-opercular activity is increased in trials with low intelligibility or errors, and that the magnitude of the cingulo-opercular response in these situations predicts performance in subsequent trials, with increases in activity associated with better performance. This mechanism is still engaged in older adults (mean age = 60; range = 50–81 years), but to a lesser extent (Vaden et al., 2015); therefore, their ability to adapt in subsequent presentations may be somehow compromised.

Hearing loss further affects cortical mechanisms for cognitive control in older adults. Hearing loss does not seem to compromise cingulo-opercular activity and adaptive control (Vaden et al., 2016). However, experiments by Erb and collaborators show that hearing loss has effects on the activation of the insula while listening both in quiet and in noise (Erb and Obleser, 2013; Erb et al., 2013). In these studies, younger (mean age = 26; range = 22–31 years) and older adults (mean age = 67; range = 56–77 years) activate the anterior insula in adverse listening conditions. However, with higher degrees of hearing loss (tested range = 5–43 dB HL), higher insula activations were observed during clear than degraded speech, demonstrating that hearing loss alters the amount of cognitive resources deployed for speech understanding (Rudner et al., 2009; Stenfelt and Ronnberg, 2009). It should be highlighted that in the experiment of Erb and Obleser (2013), speech was presented at an audible level for each participant. Hearing loss in older also adults modulates the amount of neural activity in STG as a function of the grammatical complexity of the stimuli (Peelle et al., 2011). Together, these pieces of evidence suggest that sensory loss has an impact on the neural resources used for cognitive control, and not only affects the ability to process the perceptual aspects of the speech signal. These central processing effects are unlikely to be reverted with amplification, calling for more rounded cognitive and audiological interventions in those with hearing loss.

Older adults also seem to struggle in suppressing irrelevant information not only from the auditory signal, but also from other sensory systems (Kuchinsky et al., 2012; Vaden et al., 2015, 2016), which is in turn associated with less supression of activity in sensory cortices, but also more extensive activations in prefrontal and parietal regions (Nielson et al., 2002; Gazzaley and D'Esposito, 2007; Turner and Spreng, 2012). Whereas younger adults (<40 year old) suppressed visual cortex activity when performing an auditory task, older adults (>61 years old) synchronously activated both visual and auditory cortices, failing to suppress irrelevant visual activity (Kuchinsky et al., 2012; Vaden et al., 2015, 2016). Importantly, reducing stimulus integrity had an independent but spatially similar effect to that of aging (Kuchinsky et al., 2012). This similarity in the effects of perceptual degradation and aging suggests that neural mechanisms used for challenging listening are always deployed in older adults, exacerbating the effects of noise and making all listening effortful. In addition, hearing loss further contributes to the detrimental effect of aging. Adults with hearing loss (mean age = 66; range = 45–78 years; PTA 38.4 dB HL) show less suppression of activity in occipital regions during listening than participants with less hearing loss or normal hearing (mean age = 62; range = 53–71 years; PTA 19.2 dB HL; Vaden et al., 2016). This difference in suppression of activity in the visual cortex was observed even when there were no significant differences in performance (participants were actively chosen to control for this).

This failure to suppress irrelevant sensory activity observed in older adults and in those with hearing loss, could be the result of having to allocate more cognitive resources for listening. This in turn reduces cognitive spare capacity (i.e., the amount of available cognitive resources) (Mishra et al., 2013, 2014; Rudner and Lunner, 2014). This reduced cognitive spare capacity will not only affect visual suppression, but also higher order language processing and any other function that relies on cognitive resources (Mishra et al., 2013, 2014; Rudner and Lunner, 2014). In support of this, aging and hearing loss result in more interference in dual-task paradigms (Tun et al., 2009), and worse comprehension of syntactically complex sentences (Wingfield et al., 2006a; Stewart and Wingfield, 2009; DeCaro et al., 2016). Pupilometry studies also show that aging and hearing loss are associated with less availability of cognitive resources. In normal hearing individuals, the pupil dilates as cognitive load increases, for example by decreasing the intelligibility of the speech signal. Older age (evaluated using a range of 45–73 years of age) and hearing loss (>25 dB HL) result in maintained pupil dilation across noise levels, indicating less release from listening effort as speech is more intelligible (Zekveld et al., 2011). Increased pupil dilation and cognitive load are in turn associated with increased activation of cortical auditory regions, but also frontal ones (Zekveld et al., 2014), supporting the idea that in older listeners with hearing loss more resources are allocated for listening in clear conditions. It is perhaps this unavailability of resources what compromises general cognitive function, accelerating decline.

### EEG studies

Another common method for the study of cortical function is EEG. This technique has poorer spatial resolution than fMRI, but excellent time resolution. Auditory stimulation results in an alteration of the encephalogram known as cortical auditory evoked potential (CAEP). In adults, the most prominent peaks are N1 (~100 ms post-stimulus onset) and P2 (~175–200 ms). Smaller peaks P1 and N2, preceding N1 and succeeding P2 respectively, are also often described (see for a review Wunderlich and Cone-Wesson, 2006). Changes in the amplitude and latency of these components reflect perceptual discrimination and processing (see Hyde, 1997; Kraus and Cheour, 2000; Wunderlich and Cone-Wesson, 2006; Sharma and Glick, 2016), making EEG an invaluable tool for the study of the effects of hearing loss and aging on cortical auditory function.

EEG results from studies of hearing loss have showed increased amplitude in the N1 and P2 components of the CAEP in individuals with hearing loss (compared to control groups with normal hearing; Tremblay et al., 2003; Harkrider et al., 2006; Bertoli et al., 2011; Campbell and Sharma, 2013; but see Wunderlich and Cone-Wesson, 2006). The amplitude and latency of the P2 component is positively correlated with speech-in-noise thresholds, and P2 amplitude is also positively correlated with hearing thresholds at high frequencies (Campbell and Sharma, 2013). These results suggest that the greater the degree of hearing loss and the difficulty to understand speech, the larger and more sluggish the cortical response.

Aging also has effects on the CAEP, increasing N1 and P2 amplitude, and P2 latency (Pfefferbaum et al., 1980; Anderer et al., 1996; Tremblay et al., 2002, 2003; Harkrider et al., 2005, 2006; Ceponiene et al., 2008; McCullagh and Shinn, 2013). It is interesting to note that differences between younger and older adults disappear as noise increases. McCullagh and Shinn (2013) showed that older adults (mean age = 66.4; range 62–77) have higher N1 and P2 amplitudes than younger adults (mean age = 21.4; range = 19–29) in response to an auditory oddball paradigm in quiet conditions (stimuli presented at equal sensation level for all participants). As noise was introduced in the stimuli, amplitude of the N1 and P2 was maintained in the younger group, but decreased in the group of older adults. These results can be interpreted as older adults having to deploy compensatory mechanisms while listening in quiet, as seeing in the fMRI studies described above, but not being able to deploy these mechanisms while listening in challenging conditions.

The EEG evidence reviewed above shows aging and hearing loss affecting the CAEP in the same direction. It has been suggested that these effects on the CAEP reflect inefficient cortical processing in response to a degraded signal (Harkrider et al., 2005; Ross et al., 2007). To address this issue, Harkrider et al. (2006) tested whether the effects of aging and hearing loss on the CAEP disappeared by increasing the audibility of the stimuli. Behavioral differences driven by age and hearing loss disappeared, as well as the effect of aging on the CAEP, but there was no change on the hearing loss effect on the cortical response. This highlights that the effects of age and hearing loss, despite modifying the CAEP in the same direction, could be of different nature, and thus may need different treatment strategies. These results also support the evidence obtained with MRI, which suggests that hearing loss results in cortical reorganization, demonstrating that the effects of hearing loss on cortical responses are not only a consequence of degradation of the signal or increased effort. In support of this reorganization, source localization reveals a reduction in activation in temporal cortical regions, and recruitment of frontal areas in hearing impaired individuals (~40 dB HL at 4 KHz; Campbell and Sharma, 2013). This cortical reorganization hypothesis is also in agreement with results of a magnetoencephalography (MEG) study by Dietrich et al. (2001), who showed that the group of neurons that is usually responsive to the lost frequencies starts responding to adjacent tone frequencies when there is hearing loss.

An interesting issue to consider is how much the effects that we observe in cortical responses are due to differences or compensatory mechanisms that arise in subcortical processing stages. From studies in humans and animals, it is known that both aging and hearing loss affect the auditory brainstem response (ABR), resulting in elevated thresholds and reduced amplitudes (Boettcher, 2002 for a review). We are just beginning to understand how these subcortical effects modulate cortical processing and how they are also regulated by cortical top-down signals. An excellent example of this interaction is the recent efforts to characterize the effects of hidden hearing loss in humans, which can also contribute to explaining why older adults with normal audiograms have trouble with speech perception in noise. Animal studies have revealed that noise exposure and aging can produce cochlear neuropathy without causing hair cells loss, and without affecting an individual's ability to detect sounds, resulting in “hidden hearing loss” (Kujawa and Liberman, 2009; Schaette and McAlpine, 2011; Furman et al., 2013; Plack et al., 2014; Viana et al., 2015). This is due to damaged high-threshold, medium- and low-spontaneous rate auditory nerve fibers, which are thought to encode acoustic information at medium to high levels, and when signal to noise ratio is poor (Kujawa and Liberman, 2009; Furman et al., 2013). Post-mortem histopathological analysis has shown that this type of damage exists in human adults with no history of hearing problems and with no apparent cochlear damage, likely contributing to difficulties while listening in challenging conditions (Viana et al., 2015), but without consequences on conventional audiograms. In an elegant combination of behavioral and electrophysiological techniques, Bharadwaj et al. (2015) investigated whether potential behavioral and physiological effects of hidden hearing loss could affect cortical processing. Using experimental conditions that were more likely to evoke recruitment of fibers that are more vulnerable to neuropathy, including high sound levels, off-frequency maskers, and shallow modulation depths, they found a correlation between behavioral and electrophysiological measurements of temporal coding fidelity. They further showed that poor subcortical encoding was associated with poor cortical sensitivity in interaural time differences. However, none of these measurements were related to hearing thresholds. In short, they demonstrated that effects that arise at subcortical processing stages are reflected in cortical responses, even in the absence of peripheral damage. Furthermore, this effect was found in younger adults (21–39 years of age) who reported no hearing problems and had normal audiograms (<15 dB HL), so it is likely to be worse in older adults, and a contributor to the effects of aging on listening difficulties despite normal audiograms.

Not only compromised subcortical processing is reflected on cortical responses, but top-down effects from cortical areas can also modulate subcortical stages. Sorqvist et al. (2012) observed that ABRs were modulated by working memory load, suggesting dynamic interactions between top-down and bottom-up mechanisms, e.g., cortical regions that control attention allocation regulating subcortical gating. These results show that we need to consider the nervous system as a whole, and not just investigate processing in isolated areas. More studies integrating comprehensive behavioral measurements of auditory processing and cognition, combined with human neuroscience techniques recording activity at all processing stages, will give us a better picture of how aging and hearing loss affect auditory function.

## Remaining questions and future directions

1. *What is the relationship between cognitive decline and the effects of hearing loss and aging on cortical structure and function?* The evidence reviewed in this paper shows that age and hearing loss cause atrophy in auditory regions of the human brain. This atrophy is correlated with the recruitment of compensatory mechanisms for auditory and language processing. Future research needs to focus on determining how this compensatory recruitment relates to cognitive decline.
2. *Does hearing loss result in structural changes beyond the STG?* Many studies have characterized the effects of aging on the structure of the human brain, and in particular in the prefrontal cortex. However, it is not clear whether hearing loss also causes damage in regions beyond those involved in auditory processing. Clarification of this is required for a full understanding of the interaction between hearing loss and aging. One of the first steps to achieve this is a more detailed characterization of participants' hearing abilities in the studies of aging.
3. *What is the effect of hearing loss and aging on cortical network dynamics?* So far, most studies have focused on the effects of these two factors on the function and structure of specific brain regions. Yet, cortical regions work as part of functional and structural networks, and effects on one node could affect the dynamics of the whole network. In the coming years, the field needs to investigate how these factors, in isolation and combined, influence network dynamics and what treatments are available to avoid the behavioral consequences of altered functions.
4. *Are the cortical effects of hearing loss correlated with its duration?* This is a question that has not been fully addressed in the literature, with many studies lacking information about duration of hearing loss and age of onset. Knowledge about these effects are valuable for professionals working with those who have hearing loss in order to understand fully their pathology, and the possibilities for treatments.
5. *Can early intervention prevent the effects of hearing loss on brain structure and function, and does this result in preservation of cognitive abilities?* Many of the effects of hearing loss were found with mild losses and at early stages. Therefore, an interesting topic for future research would be to determine if these can be prevented or reversed with early intervention. It is unlikely that amplification can revert the atrophy effects of hearing loss. However, early intervention may prevent them and avoid functional loss. If such an opportunity exists, and this in turn protects from cognitive decline, we need to direct our efforts into practical implementations.

## Conclusions

The evidence discussed here suggests that atrophy of cortical auditory regions is present in hearing loss and older age, potentially compromising auditory processing. In addition, due to peripheral damage, the auditory signal will be poor and degraded. In these situations, a stronger reliance on cognitive resources is necessary in order to achieve successful auditory perception, even in quiet conditions. This is supported by studies of speech perception, where there is additional recruitment of frontal cortical regions when there is damage in auditory areas, which in turn predicts behavioral performance. As a consequence, cognitive load is constantly high, deeming all listening effortful, and reducing the amount of spare cognitive capacity for other tasks (resulting in poor performance in diagnostic tests of cognitive function; Lin and Albert, 2014; Rudner and Lunner, 2014). The cortical mechanisms deployed to aid normal listening are similar to those usually engaged for listening in effortful conditions, including engagement of the saliency network, adaptive control and re-allocation of attention. The problems arise when listening conditions are challenging, and cognitive resources are no longer enough. This constant effortful listening and reduced cognitive spare capacity could be what accelerates cognitive decline in older adults with hearing loss.

In several of the studies reviewed above, hearing loss and aging have similar detrimental effects on cortical processing. However, in some situations hearing loss and aging had effects that could be dissociated (e.g., Harkrider et al., 2006; Vaden et al., 2016), suggesting more than one mechanism for impairment of cortical processing, but also more avenues for treatment. Improved understanding of the independent effects of aging and hearing loss will help us in designing successful interventions. Above all, it is important that future research evaluates whether early audiological interventions, combined with cognitive assessments, can prevent the consequences of hearing loss in brain function and structure, and reduce cognitive decline.

## Author contributions

The author confirms being the sole contributor of this work and approved it for publication.

## Funding

Funding from the Linnaeus Centre HEAD, The Swedish Research Council (grant number: 2007-8654), Action on Hearing Loss (Project 598), and the Economic and Social Research Council of Great Britain (Grant RES-620-28-0002).

### Conflict of interest statement

The author declares that the research was conducted in the absence of any commercial or financial relationships that could be construed as a potential conflict of interest.
